# Early mortality and functional outcome after acute stroke in Uganda: prospective study with 30 day follow-up

**DOI:** 10.1186/s40064-015-1252-8

**Published:** 2015-08-25

**Authors:** Jane Nakibuuka, Martha Sajatovic, Joaniter Nankabirwa, Charles Ssendikadiwa, Anthony J. Furlan, Elly Katabira, James Kayima, Nelson Kalema, Jayne Byakika-Tusiime, Edward Ddumba

**Affiliations:** Department of Medicine, School of Medicine, Makerere University College of Health Sciences, P.O. Box 7051, Kampala, Uganda; Neurological and Behavioral Outcomes Center, University Hospitals Case Medical Center, 11100 Euclid Avenue, Cleveland, OH 44106 USA; Department of Medicine, Mulago National Referral Hospital, P.O. Box 7051, Kampala, Uganda; University Hospitals Case Medical Center, Neurological Institute Case Western Reserve University, 11100 Euclid Avenue, Cleveland, OH 44106 USA; Department of Epidemiology and Biostatistics, School of Public Health, Makerere University College of Health Sciences, P.O. Box 7072, Kampala, Uganda; Department of Medicine, St Raphael of St Francis Nsambya Hospital, Nkozi University, P.O. Box 7146, Kampala, Uganda

**Keywords:** Death, Function, Outcome, Stroke

## Abstract

Identification of early outcomes post stroke and their predictors is important in stroke management strategies. We prospectively analysed 30-day outcomes (mortality and functional ability) after stroke and their predictors among patients admitted within 7 days post event to a national referral hospital in Uganda. This was a prospective study of acute stroke patients consecutively enrolled between February and July 2014. Social demographics, clinical, laboratory, imaging characteristics, outcomes (all through 30 days), time of death were assessed using standardised questionnaires. Multiple regression was used to analyse the independent influence of factors on outcomes. Of 127 patients, 88 (69.3 %) had ischemic stroke and 39 (30.7 %) had hemorrhagic stroke. Eight (6.3 %) died within 7 days, 34 (26.8 %) died within 30 days, with 2/3 of deaths occurring in hospital. Two were lost to follow up. Of 91 survivors, 49 (53.9 %) had satisfactory outcome, 42 (46.1 %) had poor functional outcome. At multivariate analysis, independent predictors of mortality at 30 days were unconsciousness (GCS <9), severe stroke at admission and elevated fasting blood sugar. None of the patients with functional independence (Barthel index ≥60) at admission died within 30 days. Inverse independent predictors of satisfactory outcome at 30 days were older age, history of hypertension and severe stroke at admission. Acute stroke patients in Uganda still have high rates of early mortality and poor functional outcomes. Independent predictors of mortality and poor functional outcome were severe stroke at admission, unconsciousness, high fasting blood sugar, old age and history of hypertension.

## Background

Stroke is emerging as a leading cause of preventable death and disability worldwide (Feigin et al. 2009, 2014; Dalal et al. 2008; Dalal and Bhattacharjee 2007). Many prospective studies based on hospital series have been reported (Sheikh et al. 1983; Smith et al. 2010; Weimar et al. 2002; Jongbloed 1986; Daverat et al. 1991; Mudzi et al. 2012; Heuschmann et al. 2004; Counsell and Dennis 2001), but little is known about 30 day case fatality and functional outcome after stroke in developing countries that currently account for 85 % of global deaths from stroke (Feigin et al. 2003, 2009, 2014; Dalal et al. 2008). Multiple characteristics have been shown to predict early mortality and dependence after stroke such as age, type of stroke, side and site of the lesion, level of consciousness, severity of neurological impairment and disability at baseline, medical risk factors (hypertension, diabetes), premorbid state, fever, blood pressure at baseline, previous stroke (Sheikh et al. 1983; Counsell and Dennis 2001; Greer et al. 2008; Whiteley et al. 2009; Hier and Edelstein 1991; Kwakkel et al. 1996).

In Uganda, the only study in this area (Kwarisiima et al. 2014) was restricted to 30 day case fatality and its prediction but did not consider functional outcome at 30 days post stroke. Severity of neurological impairment and baseline functional disability which have been shown to predict early mortality and functional outcome post stroke (Counsell and Dennis 2001) were also not assessed in this previous study. Identification of early outcomes post stroke and their predictors are important in stroke management strategies, especially in resource limited settings. The purpose of this study was to assess 30 day mortality and functional status after acute stroke and their prediction among patients admitted within 7 days post stroke.

## Methods

### Study area and setting

A prospective hospital-based study was conducted over a 6 months period between February and August 2014 at Mulago national referral and teaching hospital. It is located in the capital city Kampala and has an estimated 1500 hospital beds. The hospital’s accident and emergency unit admits on average five patients per week with acute stroke. The hospital has no stroke unit. Stroke patients are admitted to the general neurology and neurosurgery wards, and the general intensive care unit.

### Recruitment and enrolment

During the study period, 139 patients presented to Mulago hospital’s accident and emergency unit with neurologic deficits suggestive of acute stroke that occurred within the previous 7 days. Stroke was defined according to the criteria of the World Health Organisation as sudden onset of focal and at times global neurological deficits, with symptoms lasting more than 24 h or leading to death, with no apparent cause other than that of vascular origin (WHO MONICA 1988). Brain computerised tomography (CT) confirmed stroke and classification of stroke subtypes was done using the Trial of ORG 10172 and medical disability guidelines for ischemic and hemorrhagic stroke respectively (Adams et al. 1993; Reed group 1991). Two patients with suspected stroke died within a few hours of admission before a CT scan could be performed to confirm stroke. Of 137 patients that had non-contrast CT scan done, 2 patients with normal findings on both the initial and repeat CT scan (performed on day 7 from stroke onset) and 6 patients with non-stroke pathology on CT were excluded from the study. Of 129 patients with stroke confirmed on brain CT scan, 127 were recruited consecutively into the study upon provision of written informed consent to participate, while two patients with hemorrhagic stroke died before they and/or relatives could provide consent. The first choice of proxy was the spouse, live in companion, followed by a daughter/son (18 years or older), parent, sibling, or close friend of the patient. Each patient was followed for 30 days from stroke onset.

### Study procedures

An interviewer assessed selected social demographic characteristics including age, sex, tribe, district, religion, highest level of education, marital status, occupation, date of stroke onset and time to hospital presentation from stroke onset. We also evaluated history of pre-existing stroke risk factors including diabetes mellitus, hypertension, hyperlipidemia, smoking, harmful alcohol consumption, heart disease, prior stroke or transient ischemic attack (TIA). A comprehensive clinical assessment at admission included general and cardiovascular examination, measurement of waist and hip circumferences to determine waist-hip ratio, blood pressure, and neurological examination including initial level of consciousness using the Glasgow Coma Scale (GCS) (Teasdale and Jennett 1974), severity of stroke using the Scandinavian stroke scale (SSS) (Scandinavian Stroke Study Group 1985). Functional ability in activities of daily living was assessed using the Barthel index (BI) (Collin et al. 1988). Laboratory investigations included fasting lipid profile [total cholesterol, low density lipoprotein (LDL), high density lipoprotein (HDL), and triglycerides], complete blood count, fasting blood sugar, erythrocyte sedimentation rate (ESR), rapid plasma reagin (RPR) and HIV test obtained after an 8 h fast.

### Thirty days follow up

Study participants were transferred to the general neurology unit within 24 h of arrival to the accident and emergency unit. On these units, they were managed by medical officers, internal medicine physicians, neurologists, general nurses and auxiliary staffs. The standard of care for acute stroke at Mulago Hospital is a modification of the American Heart Association/American stroke association (AHA/ASA) guidelines (Adams et al. 2007; Broderick et al. 2007). This includes supportive treatment to ensure a patent airway, good oxygen saturation (target >92 %), haemodynamic stability, temperature and glycaemic control, hydration, nutrition and measures to prevent pressure sores and deep venous thrombosis. Antihypertensive drugs (labetalol and hydralazine) are used when blood pressure exceeds 160/100 and 180/105 mmHg for haemorrhagic and ischemic stroke respectively. Among patients with ischemic stroke, antiplatelet drugs including aspirin and clopidogrel, and statins are administered. Rehabilitation includes physiotherapy, occupational, speech and language therapy. Patients that require mechanical ventilation are admitted to the general intensive care unit. Recombinant tissue plasminogen activator is not a routine part of ‘standard of care’ in Uganda.

Each patient was followed for 30 days from stroke onset until either death at the hospital or discharge. Discharged patients were scheduled for a neurology outpatient clinic review that coincided with the 30-day follow up. Prior to the 30 day review date, patients were called up weekly to assess general clinical status. Date of death was obtained for those that died before 30-day follow-up. The Barthel index assessment was done at 30-day follow-up in the neurology clinic. Individuals unable to come to the neurology clinic had the 30-day follow-up conducted in their home.

### Outcome measures

Thirty days mortality and functional status as measured by the Barthel Index were the primary outcomes of interest in this study. The Barthel index reflects functional consequences for daily activities that are immediately important to a patient post stroke (Collin et al. 1988) including feeding, dressing, mobility (walking on a level surface and ascending/descending stairs or an incline), and personal hygiene (bathing, grooming, toileting, and control of bodily functions). The Barthel Index is scored on a total scale of 0–39 (total functional dependence), 40–59 (partially dependent), 60–84 (Independent) and 85–100 (total functional independence). In this study, satisfactory functional outcome was defined as a score of ≥60.

### Ethical approval

The study was approved by Makerere University College of Health Sciences’ School of Medicine higher degrees research and ethics committee, Mulago national referral hospital’s research and ethics committee, and The Uganda National Council for Science and Technology.

### Data management and statistical analysis

All statistical analyses were carried out using Stata version 12.0 software (STATA Corporation, College Station, TX, USA). Univariate analysis was used to describe socio-demographic characteristics of the study participants and summarize as percentages the study outcomes. Univariate correlation of potential prognostic factors (baseline variables) and patients’ outcomes was also performed. The baseline variables included social demographics characteristics (age, sex, religion, level of education, marital status), and pre-existing risk factors for stroke (hypertension, diabetes mellitus, heart disease, current smoking, harmful alcohol consumption, prior stroke/TIA). On examination, general assessment included temperature, pulse, cardiovascular status, blood pressure, and abdominal obesity. Neurological assessment included level of consciousness using the GCS, stroke severity at admission using the SSS and functional status using the BI. Laboratory parameters included white blood cell count, ESR, fasting blood sugar, fasting lipid profile, rapid plasma reagin, and HIV serology. Imaging procedures included non-contrast brain CT, ECG, echocardiography and carotid Doppler ultrasound for patients that had abnormal cardiovascular examination.

At multivariate analysis by logistic regression, potential predictors from univariate analysis and the literature were selected. Multivariate model building using both forward and backward elimination was used to generate minimum adequate models using a 5 % significance level for hazard ratios for death and odds ratios for satisfactory outcome at 30 days after stroke. Severity of neurological impairment at baseline and age group were retained as fixed terms in the model assessing for predictors of mortality regardless of statistical significance because of their known association to 30 day mortality after stroke. Finally, cumulative survival at 30 days post stroke, estimated survival probability at 30 days from stroke onset according to stroke subtypes and stroke severity using the SSS (mild, moderate, severe) was done using the Kaplan–Meier curves

## Results

### Characteristics of the study participants

As illustrated in (Table 1), among 127 patients, 88 (69.3 %) had ischemic stroke and 39 (30.7 %) had hemorrhagic stroke. Thirty-eight patients (23 ischemic strokes, 15 hemorrhagic strokes) presented within 24 h of symptom onset and out of these, 2 patients with ischemic stroke and 3 patients with haemorrhagic stroke presented within 6 h. Of 127 patients, 68 (53.5 %) were female. The age range was 19–99 years with median 60 (IQR 49–75). There were similar numbers of patients <51 years (29.9 %) and >71 years (29.1 %). More than half of the patients were unemployed and had either never attended school or attained a primary level of education. Out of 127 patients, only 6 were HIV positive. Fifty-three patients had an abnormal cardiovascular assessment with atrial fibrillation on ECG in 6 patients and cardiovascular source of emboli on echocardiography and carotid Doppler ultrasound scan in 7 patients.

**Table 1:** Characteristics of the study patients

Characteristics	All participants n (%)	Ischemic stroke participants n (%)	Hemorrhagic stroke participants n (%)
Sex			
Male	59 (46.5)	40 (45.5)	19 (48.7)
Female	68 (53.5)	48 (54.5)	20 (51.3)
Age group (years)			
<51	38 (29.9)	28 (31.8)	10 (29.3)
51–60	26 (20.5)	14 (15.9)	12 (30.8)
61–70	26 (20.5)	20 (22.7)	6 (15.4)
≥71	37 (29.1)	26 (29.6)	11(28.2)
Median age (IQR)	60 (49–75)	62 (49–74.5)	60 (50–75)
Religion			
Catholic	42 (33.1)	29 (32.9)	13 (33.3)
Protestant	48 (37.8)	33 (37.5)	15 (38.5)
Islam	29 (22.8)	21 (23.9)	8 (20.5)
Other	8 (6.3)	5 (5.7)	3 (7.7)
Highest level of education			
Never attended school	11 (8.7)	7 (7.9)	4 (10.3)
Primary	60 (47.2)	43 (48.9)	17 (43.6)
Secondary	38 (29.9)	26 (29.5)	12 (30.8)
Tertiary	18 (14.2)	12 (13.6)	6 (15.4)
Marital status			
Single	8 (6.3)	5 (5.7)	3 (7.7)
Separated or divorced	24 (18.9)	17 (19.3)	7 (17.9)
Widowed	29 (22.8)	19 (21.6)	10 (25.6)
Married	66 (52.0)	47 (53.4)	19 (48.7)
Employment level			
Un-employed	69 (54.3)	48 (54.5)	21 (53.8)
Casual employment	44 (34.7)	28 (31.8)	16 (41.0)
Professional employment	14 (11.0)	12 (13.6)	2 (5.1)
HIV status			
Negative	121 (95.3)	85 (96.6)	36 (92.3)
Positive	6 (4.7)	3 (3.4)	3 (7.7)

### Death and functional outcome at thirty days after stroke

**Fig. 1:**
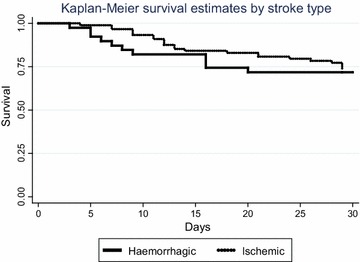
Thirty day survival after stroke, by stroke subtype

Out of 127 patients, 2 were lost to follow up, 8 (6.3 %) died within 7 days and 34 (26.8 %) died within 30 days from stroke onset (11 out of 39 haemorrhagic strokes (28.2 %), 23 out of 88 ischemic strokes (26.1 %) (Fig. 1). Overall, in-hospital death occurred in 23 of 127 patients (Hemorrhagic 28.2 % and ischemic 13.6 %, P = 0.049). The mean survival time was 25.7 days (SD 8.1) and the average length of hospital stay was 8.9 days (SD 5.8). At 30 days from stroke onset there were 91 survivors (71.7 %) and among them, 49 (53.9 %) had satisfactory outcome (28 totally independent and 21 independent) and 42 (46.1 %) had poor functional outcome (14 partially dependent and 28 totally dependent).

### Predictors of mortality at 30 days from stroke onset

As illustrated in (Table 2), 28 independent variables were tested against 30 day mortality at univariate analysis. A full correlation matrix is available on request. There was a significant difference between participants who died and those who survived to 30 days from stroke onset with respect to age ≥71 years (survivors less likely to be old, p = 0.023), fever (survivors less likely to have fever, p < 0.003), initial level of consciousness (less altered consciousness in survivors, p < 0.001), severity of stroke at admission (less severity in survivors, p < 0.005), disability at admission (less disability in survivors, p = 0.026), and high fasting blood glucose (less elevated glucose in survivors, P < 0.001).

**Table 2:** Univariate correlation of 28 independent variables with mortality or satisfactory outcome (dependent variables) or both

Independent variables associated with at least one dependent variable, P < 0.05
Social demographics
Age
Marital status^a^
Physical examination and neurological assessment
Fever (≥37.5 °C)^a^
Initial level of consciousness^a^
Stroke severity at admission
Disability at admission
Laboratory investigations
Fasting blood sugar
White blood cell count
Independent variables not associated with at least one dependent variable, P > 0.05
Social demographics
Sex^a^
Education^a^
Pre-existing stroke risk factors
Smoking^a^
Alcohol consumption^a^
History of hypertension^a^
History of diabetes mellitus^a^
History of heart disease^a^
History of stroke/transient ischemic attack^a^
Physical examination and neurological assessment
Irregular pulse^a^
Blood pressure at admission (≥140/90 mmHg)^a^
Abnormal cardiovascular examination^a^
Abdominal obesity^a^
Laboratory investigations
Total cholesterol
Low density lipoprotein cholesterol
High density lipoprotein cholesterol
Triglycerides
Rapid plasma reagin^a^
HIV^a^
ESR^a^
Imaging investigations (CT scan)
Stroke subtype^a^

^a^Dichotomous variables, *HIV* Human Immunodeficiency Virus, *ESR* Erythrocyte Sedimentation Rate

**Fig. 2:**
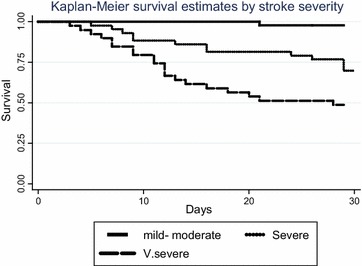
Thirty day survival after stroke, by degree of stroke severity at admission

As illustrated in (Table 3) in multivariate analysis, initial level of consciousness HR 4.4 (1.2–16.9), stroke severity at admission HR 25.5 (2.7–238) (Fig. 2) and fasting blood sugar HR 3.7 (1.1–12.4) were independent predictors of mortality at 30 days in this series. Fever was significantly associated with 30 days mortality at univariate analysis in this study but the sample size was small to allow for further analysis.

**Table 3:** Predictors of mortality at 30 days after acute stroke

Variable	Dead n/N (%)	Unadjusted HR 95 % CI	P value	Adjusted HR 95 % CI	P value
Sex					
Male	13/59 (22.0)	1			
Female	21/68 (30.9)	1.6 (0.7–3.6)	0.263		
Age group (years)					
<51	7/38 (18.4)	1		1	
51–60	4/26 (15.4)	0.8 (0.2–3.1)	0.752	1.4 (0.2–7.7)	0.713
61–70	7/26 (26.9)	1.6 (0.5–5.4)	0.421	1.4 (0.3–6.3)	0.660
≥71	16/37 (43.2)	3.4 (1.2–9.6)	0.023	3.8 (0.9–15.7)	0.069
History of stroke/TIA					
No	7/35 (20.0)	1			
Yes	27/90 (30.0)	1.7 (0.7–4.4)	0.263		
Don’t know	0/2 (0)	N/A			
History of hypertension					
No	13/49 (26.5)	1			
Yes	21/78 (26.9)	1.02 (0.5–2.2)	0.961		
Febrile (≥37.5 °C)					
No	27/118 (22.9)	1			
Yes	7/9 (77.8)	11.8 (2.3–60.1)	0.003		
Hypertension (≥140/90 mmHg)					
No	11/30 (36.7)	1		1	
Yes	23/97 (23.7)	0.5 (0.2–1.3)	0.165	0.4 (0.1–1.3)	0.135
Initial level of consciousness					
GCS ≥9	21/104 (20.2)	1		1	
GCS <9	13/23 (56.5)	5.1 (1.9–13.3)	0.001	4.4 (1.2–16.9)	0.029
Stroke severity at admission					
Mild–Moderate (30–58)	1/45 (2.2)	1		1	
Severe (15–29)	13/43 (30.2)	19.1 (2.4–153.6)	0.006	15.3 (1.7–136)	0.014
Very severe (0–14)	20/39 (51.3)	46.3 (5.8–370.4)	<0.0001	25.5 (2.7–238)	0.004
Fasting blood sugar					
Normal	9/65 (13.9)	1		1	
IGT	8/26 (30.8)	2.7 (0.9–8.2)	0.067	2.2 (0.6–8.1)	0.249
High	17/36 (47.2)	5.6 (2.1–14.6)	<0.0001	3.7 (1.1–12.4)	0.031
Stroke subtype					
Ischemic	23/88 (26.1)	1		1	
Hemorrhagic	11/39 (28.2)	0.9 (0.4–2.0)	0.808	1.8 (0.6–5.7)	0.294

### Predictors of satisfactory outcome at 30 days from stroke onset

Multiple potential predictors of satisfactory outcome were selected at univariate analysis as was done for 30-day mortality as illustrated in (Table 2). At univariate analysis there was a significant difference between participants who had satisfactory outcome and those who had a poor outcome at 30 days from stroke onset with respect to age ≥71 years (survivors less likely to be older, P = 0.002), marital status (survivors likely to be married, P = 0.045), initial level of consciousness (less unconsciousness in satisfactory outcome, p = 0.010), stroke severity at admission (lower severity in satisfactory outcome, P < 0.0001), fasting blood sugar (less elevated in satisfactory outcomes, P = 0.001), and total white blood cell count (less elevated in satisfactory outcome, P = 0.007).

As presented in (Table 4), in multivariate analysis, satisfactory outcome at 30 days was inversely correlated with age ≥71 year OR 0.2 (95 % CI 0.04–0.7), history of hypertension OR 0.3 (95 % CI 0.1–0.9) and stroke severity OR 0.01 (95 % CI 0.002–0.09).

**Table 4:** Predictors of satisfactory outcome at 30 days after acute stroke in 127 patients

Variable	Satisfactory outcome n/N (%)	Unadjusted OR 95 % CI	P value	Adjusted OR 95 % CI	P value
Sex					
Male	28/59 (47.5)	1			
Female	21/68 (30.9)	0.5 (0.2–1.02)	0.057		
Age group (years)					
<51	21/38 (55.3)	1		1	
51–60	12/26 (46.2)	0.7 (0.2–1.9)	0.475	0.4 (0.1–1.8)	0.244
61–70	9/26 (34.6)	0.4 (0.4–1.2)	0.107	0.6 (0.1–2.4)	0.434
≥71	7/37 (18.9)	0.2 (0.1–0.5)	0.002	0.2 (0.04–0.7)	0.015
Education					
None and primary	24/71 (33.8)	1			
Secondary and tertiary	25/56 (44.6)	1.6 (0.8–3.2)	0.214		
Marital status					
Not married	18/61 (29.5)	1			
Married	31/66 (46.9)	2.1 (1.01–4.4)	0.045		
History of stroke/TIA					
No	15/35 (42.9)	1			
Yes	33/90 (36.7)	0.8 (0.3–1.7)	0.523		
Don’t know	1/2 (50.0)	1.3 (0.1–23.1)	0.843		
History of hypertension					
No	24/49 (48.9)	1		1	
Yes	25/78 (32.1)	0.5 (0.2–1.02)	0.058	0.3 (0.1–0.9)	0.033
Febrile					
No	47/118 (39.8)	1			
Yes	2/9 (22.2)	0.4 (0.1–2.2)	0.308		
Hypertension (>140/90 mmHg)					
No	11/30 (36.7)	1			
Yes	38/97 (39.2)	1.1 (0.5–2.6)	0.805		
Initial level of consciousness					
GCS ≥9	46/104 (44.2)	1		1	
GCS <9	3/23 (13.0)	0.2 (0.1–0.7)	0.010	0.3 (0.04–2.3)	0.261
Stroke severity at admission					
Mild and moderate	35/45 (77.8)	1		1	
Severe	12/43 (27.9)	0.1 (0.04–0.3)	<0.0001	0.06 (0.02–0.2)	<0.0001
Very severe	2/39 (5.1)	0.02 (0.003–0.07)	<0.0001	0.01 (0.002–0.09)	<0.0001
Fasting blood sugar					
Normal	33/65 (50.8)	1			
Impaired glucose tolerance	10/26 (38.5)	0.6 (0.2–1.5)	0.290		
High	6/36 (16.7)	0.2 (0.1–0.5)	0.001		
White blood cell count					
Low	9/13	1			
Normal	37/97	0.1 (0.1–0.9)	0.042		
High	3/17	0.1 (0.01–0.5)	0.007		
Stroke subtype					
Hemorrhagic	13/39 (33.3)	1		1	
Ischemic	36/88 (40.9)	1.4 (0.6–3.1)	0.419	0.4 (0.1–1.5)	0.169

## Discussion

Information of early survival and functional recovery after acute stroke and their prediction is important in stroke management strategies especially in resource limited settings.

The 30 days case fatality rate in this study was high, although considerably lower than the mortality rate of 43.8 % in a study done in the same hospital 3 years prior (Kwarisiima et al. 2014). Our findings are comparable to recent studies on stroke in Africans of 27–46 % (Kwarisiima et al. 2014; Ogun 2004; Ogun et al. 2005; Connor et al. 2007a, b; Walker et al. 2011) but higher than studies in developed countries with 30 days stroke mortality rates less than 15 % (Smith et al. 2010; Mudzi et al. 2012; Heuschmann et al. 2004; Saposnik et al. 2008; O’ Donnell et al. 2010). The overall in-hospital death in this study was comparable to studies in national hospitals in Africa (Ogun et al. 2005; Ojini et al. 2008) but lower than in-hospital stroke mortality in North America (Smith et al. 2013). Thrombolysis and organized multidisciplinary stroke units widely available in developed countries can reduce mortality from stroke (Feigin et al. 2009; Langhorne 2007; Connor et al. 2009; Thijs et al. 2009; Carter et al. 2006; Ayis et al. 2013). Better management of pre-stroke risk factors, particularly the use of ACE inhibitors (Selim et al. 2005; Álvarez-Sabín et al. 2007), aspirin (Wilterdink et al. 2001; Ovbiagele et al. 2008) and the wider use of pre-stroke statins (Yoon et al. 2004) may prevent catastrophic stroke that is common in the African setting (Feigin et al. 2009). In addition, most of the studies in developed countries are community based. In Africa on the other hand, the high case fatality has been blamed on limited health care access, delayed presentation to hospital, and a shortage of adequately trained professionals to provide acute care and rehabilitation of stroke patients (Owolabi et al. 2007) as well as lack of stroke units. Similar factors may be responsible for the poor functional outcome among the survivors seen in this study. The early recovery in activities of daily living among stroke survivors in developed countries is generally more favorable (Wade and Hewer 1987) than our findings. This study dealt with patients admitted to a national referral hospital. Hospital data on stroke are usually biased towards the more serious or complicated cases (Bamford et al. 1986). It is possible that only the most severe stroke cases end up presenting to hospital, and that this cohort is not likely to be entirely representative of all strokes in the community, given that a large number of stroke patients per year is expected in a hospital with over 1500 beds serving Kampala’s 1.6 million people, as well as an additional estimated 1.5 million from neighbouring districts and beyond. Also, only participants with onset of stroke symptoms within 7 days were recruited. While our study does not permit a causal inference regarding the relatively high mortality outcomes, it is likely that care access issues in line with limited health budgets in most of sub-Saharan Africa play a substantial role.

A positive aspect of our study findings is that compared with Kwarisiima study at the same hospital done several years prior, stroke outcomes appear to be somewhat improved (Kwarisiima et al. 2014) although this comparison can hardly be made in rational terms. In Kwarisiima study 30 % of the stroke patients were unconscious and hence more severe strokes compared to 18 % in our study, which might account for the apparent improvement. There have been recent improvements in resources available at the national referral hospital in Ugandan, including the addition of neurology specialists to the work force, visiting international neurology specialists to help train students and staff, availability of additional resource for CT scanning, and a 50 % increase in number of mechanical ventilator support devices. In addition, a trauma center with dedicated staff was opened up to deal with severely injured trauma patients that form the bulk of emergencies at the accident and emergency unit at the hospital. This ensured that non-trauma emergencies presenting to the non-trauma resuscitation room were much better attended to. Finally, a standardised triage tool was introduced at the accident and emergency unit and staff trained in emergency triage could more readily refer individuals with suspected stroke to a care setting that could potentially minimize stroke-related complications such as respiratory compromise or infection. Never the less, it is more likely that differences in case mix between 2010 and 2014 can explain statistically the differences in mortality, without underestimating the role of the improvements in stroke care.

With respect to risk factors for mortality, unconsciousness, severe stroke at admission, high fasting blood sugar are known to be predictors of early mortality after acute stroke (Counsell and Dennis 2001; Veerbeek et al. 2011) and these were independent predictors of 30 day mortality in this study. Hospital data on stroke are usually biased towards the more serious or complicated cases (Bamford et al. 1986) and this study dealt with patients admitted to a national referral hospital. Unconsciousness and severe stroke as independent predictors of early mortality were directly related to the severity of the neurological damage (Sheikh et al. 1983; Heuschmann et al. 2004). None of the patients with independent scores (BI >60) on the disability scale at baseline died at 30 days from stroke in this study in agreement with studies that found greater disability on admission to be associated with worse survival (Hier and Edelstein 1991). Fasting blood sugar as an independent predictor of 30 days mortality was in disagreement with some studies (Cztonkowska et al. 1997). Severe acute stroke has been associated with reactive hyperglycemia even in patients without a history of diabetes due to a major stress response which accounts for the worse prognosis of these patients (Gray et al. 2004). Impaired glucose metabolism, with prevalence of previously unrecognized diabetes mellitus or impaired glucose tolerance preceding stroke is also responsible for hyperglycemia after stroke (Gray et al. 2004). Risk factor screening of the general populations in limited resource settings is low.

Multivariate analysis in this study showed that satisfactory outcome was inversely associated with old age, history of hypertension, and severity of stroke at admission. Severity of stroke at admission was in agreement with multiple studies in a systematic review (Counsell and Dennis 2001), as well as increasing age as a predictor of unsatisfactory outcome (Hier and Edelstein 1991; Kwakkel et al. 1996). However, older age appeared protective for longer term survival while vascular risk factors such as hypertension appeared to have little effect (Counsell and Dennis 2001). It is possible that risk factors for stroke survival in Africa may differ from other settings. For example, older individuals may be less likely to have an extended support system of adult care givers than their younger counter parts. Perhaps in a setting where formal healthcare is scarce, family support may be more the norm and have a protective effect in stroke recovery. More information is needed, especially concerning the influence of social demographics on stroke outcome in addition to many other predictor variables (Counsell and Dennis 2001; Hier and Edelstein 1991; Kwakkel et al. 1996).

There are limitations of our study. The sample size was small hampering the analysis of some prognostic indicators. It is hospital-based and not likely to be entirely representative of strokes occurring in the community. Also the cohort was restricted by the inclusion of only acute stroke patients with symptom onset within a week. However, the study provides a preliminary database on early mortality and functional outcome which can inform stroke management strategies.

## Conclusions

Early death, relatively high in-hospital mortality, and poor functional outcome are common in Ugandans who experience acute stroke. However, compared to a previous study conducted in the same setting, stroke outcomes may be improving. Factors associated with death and functional outcome were largely non modifiable.
